# A Complete Audit Cycle Assessing Compliance in a Mid-West Ireland Psychiatry of Later Life (POLL) Setting With “Safe Prescribing and Dispensing of Controlled Drugs” Joint Guidance by the Irish Medical Council and Pharmaceutical Society of Ireland

**DOI:** 10.1192/bjo.2025.10622

**Published:** 2025-06-20

**Authors:** Stephen Killilea, Sean Crowley, Niamh McCarthy, Tom Reynolds

**Affiliations:** 1 Acute Psychiatric Unit, Ennis General Hospital, Clare, Ireland; 2 Ravenscourt, St Finbarr’s Hospital, Cork, Ireland; 3 Psychiatry of Later Life, Gort Glas, Ennis, Clare, Ireland

## Abstract

**Aims:** Controlled drugs are defined as substances, products or preparations that are either known to be or have the potential to be dangerous or harmful to human health, including being liable to misuse or cause social harm. The Misuse of Drugs Regulation 2017 came into force on 4 May 2017 and all prescribers must adhere to these regulations. The objectives of this audit were to assess, intervene and improve compliance of a POLL Community Mental Health Team (CMHT) service with the gold standard prescribing guidelines illustrated in the “Safe Prescribing and Dispensing of Controlled Drugs” Joint Guidance by the Irish Medical Council and Pharmaceutical Society of Ireland in order to allow best practices to develop in the care of patients.

**Methods:** Prescriptions required certain details to be included e.g. strength, form and quantity of medication (words and figures) to be deemed valid and compliant. During the initial stage of the audit, every patient file was reviewed and the most recent prescription checked to assess if the patient was prescribed a controlled drug since receiving treatment from the service. A data collection tool was utilised to compare each prescription to the gold standard guidelines and all omissions were recorded. The results of the initial audit were circulated to the prescribing members of the CMHT and presented at the weekly multidisciplinary team meeting. Improving inclusion of components deemed poorly compliant on the initial stage of the audit was emphasised.

**Results:** Of the 158 files initially audited, 70 patients were prescribed controlled drugs (exclusively benzodiazepines and z-hypnotic medications) by the POLL CMHT. 100% compliance was observed regarding inclusion of date, name of patient, strength of medication, dosing frequency, signature, profession and registration number of prescriber. 98% compliance was observed regarding inclusion of name of prescriber and telephone number of prescribing setting. 97% and 94% compliance was observed regarding inclusion of address of prescriber and patient respectively. 61% and 47% compliance was observed regarding inclusion of form and quantity of medication respectively. Re-audit was completed 3 months later which showed 15/70 patients had been reissued prescriptions. The compliance rate improved to 100% for all components.

**Conclusion:** Compliance greatly improved particularly regarding components of prescriptions frequently omitted during the initial audit stage i.e. form and quantity of medication. Other components were largely compliant during both stages. As a result of this audit cycle, controlled drug prescribing patterns underwent significant quality improvement and became more aligned with the gold standard.

